# Alternative sources of valine and isoleucine for prompt reduction of plasma leucine in maple syrup urine disease patients: A case series

**DOI:** 10.1002/jmd2.12327

**Published:** 2022-09-05

**Authors:** Maryam Ziadlou, Anita MacDonald

**Affiliations:** ^1^ Department of Food Science and Technology, Science and Research Branch Islamic Azad University Tehran Iran; ^2^ Dietetic Department, Birmingham Children's Hospital Birmingham UK

**Keywords:** isoleucine supplement, leucine‐free formula, metabolic crisis, MSUD, valine supplement

## Abstract

In maple syrup urine disease (MSUD), leucine (Leu) accumulation, and its metabolites cause brain toxicity, and at diagnosis rapid plasma Leu reduction is essential. Valine (Val) and isoleucine (Iso) supplements are necessary to promote anabolism and enable prompt reduction of plasma Leu. Val/Iso supplements are unavailable in Iran, so an alternative source was necessary. An emergency protocol was developed using an unconventional source of Val and Iso to prompt reduction of high plasma Leu levels during an acute metabolic crisis to prevent brain encephalopathy and neurological sequelae. Five children with classical MSUD were referred aged 1–25 months, with a prolonged high plasma Leu of more than 1500 μmol/L and acute symptoms (irritability, poor feeding, and hypotonia). Initially, breast milk/regular infant formula was stopped. Val and Iso were given in calculated amounts from a Leu‐free formula containing Iso/Val (Xleu Maxamaid, Nutricia Ltd.) to promote anabolism. It was prescribed for a controlled and limited time with a branched chain amino acid (BCAA) free formula. Frequent amino acid monitoring was conducted. Natural protein was re‐added after normalizing plasma Leu. Plasma Leu declined by a median (range) of 1677 (1501–1852) μmol/L within 3–4 days of intervention. The median follow‐up time was 24 months (range: 14–32) and patients showed improvement in motor and cognitive skills after normalizing plasma Leu (75–200 μmol/L). Most had improvement in their head circumference (*n* = 4). Due to the unavailability of individual Val/Iso supplements, a Leu‐free formula rapidly lowered plasma Leu concentrations during acute crisis, to prevent cerebral edema and brain damage in MSUD.

**SYNOPSIS**

In MSUD patients, in the absence of valine/isoleucine supplements, the calculated amount of leucine‐free formula (containing valine and isoleucine) promoted anabolism and enabled prompt reduction of plasma leucine during metabolic crisis and may prevent cerebral damage.

## 1 INTRODUCTION

Maple syrup urine disease (MSUD; OMIM# 248600) is an inherited autosomal recessive disorder caused by deficiency of branched‐chain alpha‐keto acid dehydrogenase (BCKDH) activity, resulting in impaired metabolism of branched chain amino acid's (BCAA's): leucine (Leu), isoleucine (Iso), and valine (Val). It is characterized by poor feeding, vomiting, lethargy, dystonia, and delayed development. The increase in BCAA, particularly Leu leads to inhibition of the uptake of the transport of large neutral amino acids into the brain and causes reduction in neurotransmitter synthesis and myelination.^1^, ^2^ Accumulation of alpha‐ketoisocaproic acid (aKIC) derived from Leu is the main neurotoxic metabolite and causes metabolic encephalopathy and life‐threatening brain edema within the first week of life.^2^, ^3^, ^4^, ^5^ The longer the exposure to high Leu concentrations after birth the more seriously the intelligence quotient (IQ) is affected.^4^, ^6^ In Iran, there is no comprehensive newborn screening program for detecting MSUD patients except in three centers in different provinces, so diagnosis usually follows the first clinical presentation. Due to the serious brain toxicity of high plasma Leu levels, one of the main goals of nutrition therapy is rapid reduction of plasma Leu and its metabolite, aKIC, to avoid brain encephalopathy and permanent brain damage, with maintenance of Val and Iso concentrations in the target therapeutic range. Generally, plasma Leu concentrations decrease more gradually than Val and Iso and may be resistant to decline particularly during infection or sepsis.^7^ BCAA‐free formula, and an adequate energy intake (120–140 kcal/kg per day) are necessary to support anabolism and stabilize the plasma BCAA levels.^5^ In MSUD typically plasma concentrations of Val and Iso are lower than Leu, so their normalization will occur more rapidly than Leu, when administrating BCAA‐free formula as the sole source of protein. Patients require additional Val and Iso supplements to promote anabolism, accelerating plasma Leu reduction and preventing Val and Iso deficiency.^5^, ^7^, ^8^, ^9^, ^10^ In Iran, there is no access to Val and Iso supplements, so, we report in five cases with MSUD, the use of an alternative source of Val and Iso to rescue them from brain encephalopathy at the time of presentation and as part of an emergency protocol during illness.

## 2 MATHERIALS AND METHODS

### 2.1 Study design and patients' information

This is a case series of five MSUD patients (two females, three males); all born to consanguineous parents except Patient 2. The patients were referred to the clinic by their pediatric metabolic endocrinologist from different provinces. The median (range) age of the first metabolic crisis was 6 (5–7) days and 2.6 (1.12–25) months at referral. The median (range) of plasma Leu concentration was 2800 (2277–3500) μmol/L at diagnosis and was 1880 (1520–1937) μmol/L at intervention (Table 1).

**TABLE 1:** Biochemical profiles of five MSUD patients at presentation and post intervention

Patients (N/S)	At diagnosis				Age, weight and biochemical data at intervention					Model of intervention			Plasma amino acid after intervention		
	Age (d)	Leu (μmol/L)	Allo‐IIe (μmol/L)	Seizure (frequency)	Age (d/m)	Weight (kg)	Leu (μmol/L)	Val (μmol/L)	Iso (μmol/L)	BCAA‐free formula (ml)^a^	XLeu Maxamaid (g)^b^	Duration (d)	Leu (μmol/L)	Val (μmol/L)	Iso (μmol/L)
1/boy	21	2422	42.5	0	52 d	4.600	1880	486^c^	213	433	20	3	28	29	156
2/boy	21	3305	NR	0	36 d	3.100	1902	177	41	533	20	3	159^d^	151	91
3/girl	10	2277	NR	1	66 d	3.650	1632	224	10	433	20	3	16	204	229
4/boy	12	2800	NR	2	9 m	7.700	1520	52	228	566	15	4	19	18	144
5/girl	15	3500	93.3	>10	25 m	11.900	1937	633^c^	568	540	20	3	260^d^	291	125

Abbreviations: Allo‐IIe, alloisoleucine; BCCA, branched chain amino acid; d, days; g, gram; Iso, isoleucine; Leu, leucine; m, month; ml, milliliter; MSUD, maple syrup urine disease; *N*, number; NR, not reported; S, sex; Val, valine.

^a^ Contains 2.0 g protein/100 ml.

^b^ Contains 46 mg Val and 41 mg Iso per 5 g of dry powder.

^c^ Patients 1 and 5 received 12 and 24 h BCAA‐free formula respectively as an only source of protein to have a slight reduction in Val and Iso before intervention.

^d^ Patients 2 and 5 received 1 g of protein from breast milk and rice flour pudding, respectively, 2 h before test.

### 2.2 Confirmatory tests

They were diagnosed with elevated concentrations of BCAA's (Leu, Iso, and Val), using high performance liquid chromatography (HPLC). In addition, Patients 1 and 5 had high concentrations of alloisoleucine reported by HPLC. Patients 1 and 4 had molecular confirmation of MSUD type 1a by whole‐exome sequencing test indicating BCKDHA gene mutation with a variant of c.1069C>T (p.Q357X), and c.288+1G>.

### 2.3 Clinical presentation before intervention

Two patients had severe skin lesions, three experienced seizures and high ammonium concentrations, and one had neonatal sepsis. Patients 1 and 3 had at least 26 days hospitalization, and details are shown in Table 2. They all received BCAA‐free formula with controlled and restricted intake of natural protein from regular formula, breast milk, or low protein foods. Hypotonia and dystonia were seen in all infants with a head circumference below the third percentile according to the age.

**TABLE 2:** Patients clinical presentation before and after intervention

Patients (N/S)	Age	Sex	Clinical presentation before intervention	Changes in development after intervention
1	52 d	Boy	Twenty‐seven days in hospital: with hypotonia, lethargy, poor head control, no eye contact, and no response to sound.	Head control at 4 months, rolled over at 7 months, sat unaided at 9 months, speech started at 10 months, walking at 18 months, and ball kicking and climbing stairs at 22 months. Cognitive and motor skills ability appropriate for age. Current age: 34 months.
2	36 d	Boy	Twenty‐six days in hospital: had high ammonia concentration at 7 days (116 μmol/L), respiratory arrest and cyanosis at 15 days of age supported by a ventilator for 7 days, with irritability and hypotonia. Unable to suck and move hands and arm, no response to sound.	Sucking ability improved in 7 days, smiled 10 days after intervention, moved his hands, arms, and rolled over 14 days after intervention. Able to raise head when lying on stomach at age 4.5 month, Crawled at age 5.5 months. Head control at 7 months. Sat with supporting arm at 14 months. Current age: 17 months.
3	66 d	Girl	Sixty days in hospital: sepsis at 5 days, seizure at 6 days with high ammonia concentration (138 μmol/L), experienced coma supported by a ventilator for 11 days, with hiccups, and difficulty breathing. At referral, had irritability with persistent crying, severe skin lesions and dehydration with flaky skin. There was also poor feeding and hypotonia, severe palmar grasp reflex in her fist, could not move head from side to side, severe nystagmus without smiling or eye contact.	Skin recovered at 15 days and smiling 22 days post intervention. Opened clenched fist at 43 days; head control at 9 months, rolled over by 8 months. Speech commenced at 12 months, sat unaided at 14 months, and crawled at 23 months. Nystagmus improved and head circumference increased by 12 cm over 1 year. Current age: 26 months.
4	9 m	Boy	Forty‐three days in hospital: seizures on day 4, had severe skin lesions and dehydration with flaky skin, and secondary cornea ulcer associated with low plasma Val and Iso for >1 month. Feed refusal due to mouth ulcers. Severe irritability and persistent crying, poor head control, unable to roll over or sit unaided. Had high plasma Leu for >8 months.	Skin recovered at 18 days post intervention. Eye contact improved within 5 weeks. Head control and speech commenced at 13 months, rolled over at 14 months, and sat unaided at 15 months. Crawled at 19 months, walkedat 25 months, and climbed stairs at 27 months of age. Current age: 38 months.
5	25 m	Girl	Four hospitalizations: had high ammonia concentration (202 μmol/L). Seizures and coma during infancy, supported by ventilator for 10 days, with irritability, hypotonia, no speech, Unable to crawl, walk and sit unaided, Had palmar grasp reflex in her fist.	Speech commenced 10 days post intervention aged 25 months. No hand spasm 15 days after intervention, started to pick up objects by using thumb and index finger and able to turn the pages of a book after 20 days, and bring hands together after 30 days, stood while holding on to furniture 40 days post intervention. Crawled at 34 months and could eat independently at 36 months of age. Also at 36 months, able to draw with a pencil and erase. She is walking with support and drinking water from a cup. Current age: 39 months.

Abbreviations: d, day; m, month.

### 2.4 Dietary treatment

Initially, the source of Leu in breast milk, regular infant formula, or food was stopped. Due to the inability to access Val and Iso supplements to accelerate anabolism, 15–20 g/day of a Leu‐free formula (Xleu Maxamaid, Nutricia Ltd.) was added to a BCAA‐free formula (calculation based on protein requirements [g/kg]). The Leu‐free formula contained 41 and 46 mg Iso and Val, respectively, per 5 g of dry powder. It was given for 3–4 days according to the plasma Leu concentration. The higher the plasma Leu, the more Leu‐free formula was given (Table 1). Parents were advised to administer this formula gradually (5 g powder diluted in 30 ml water hourly) to prevent a sudden rise of plasma Val and Iso levels, but to complete the recommended dosage within 3 to 4 hours. For patients with low Val and Iso levels (below 200 μmol/L) but Leu concentrations over 1500 μmol/L, the intervention by Leu‐free formula was started promptly (Patients 2, 3, and 4). If plasma Val and Iso were more than 400 μmol/L, BCAA‐free formula only was given orally or by tube for the first 12–24 h to modify plasma levels of Val and Iso before commencement of their supplementation (Patients 1 and 5). The plasma amino acid profile was measured (3–4 days post intervention) by HPLC. After normalizing the plasma Leu concentrations below 200 μmol/L, the calculated amount of Leu from natural protein (regular formula, breast milk or foods) was re‐added to their diet immediately to provide the Leu requirement. For patients older than 6 month the parents were advised to use a low protein Iranian recipe book that had been developed by the Center for Diseases Control and Prevention Ministry of Health and Medical Education of Iran.^11^ Patients leucine tolerance was 30‐78 mg/kg/day and was determined according to weight, age, and stability of plasma leucine levels. The natural protein content was estimated from leucine tolerance (1 g protein is equivalent to 60 mg leucine).^8^, ^12^ Omega 3 fatty acids commonly low in protein restricted diets used in IMD, were supplemented, using FAO requirements as a guide.^13^, ^14^ Children aged from 6 to 12 months (Patients 1–3) were prescribed 40 mg/day docosahexaenoic acid (DHA) and from the age of 1 year (except Patient 5) 95 mg/day DHA and 150 mg/day of eicosapentaenoic acid (EPA). Patient 5 received supplementation after the first visit at 25 months of age.^14^, ^15^, ^16^

### 2.5 Patient follow up

Weight, height, and head circumference of children were measured at each clinic visit. Infants were evaluated daily, weekly, or twice monthly for a minimum of 1 year to ensure sufficient amounts of essential amino acids were provided according to the GMDI/SERC monitoring MSUD guideline.^9^, ^17^ Parents were trained about the signs and symptoms of BCAA amino acid deficiencies. They were advised to send photographs of any possible sign of skin rashes or any other redness on the body, anal region, back of the ear and around the chin daily during the intervention and follow‐up period.

### 2.6 Ethical consideration

All procedures were in accordance with World Medical Association Declaration of Helsinki.^18^ All parents gave written informed consent and agreed that their children's medical data could be published anonymously.

### 2.7 Statistical analysis

Data were summarized with descriptive statistics using median, and range to describe the general data. Pearson correlation coefficient was used to calculate the correlation between plasma Leu and head circumference.

## 3 RESULTS

**FIGURE 1:**
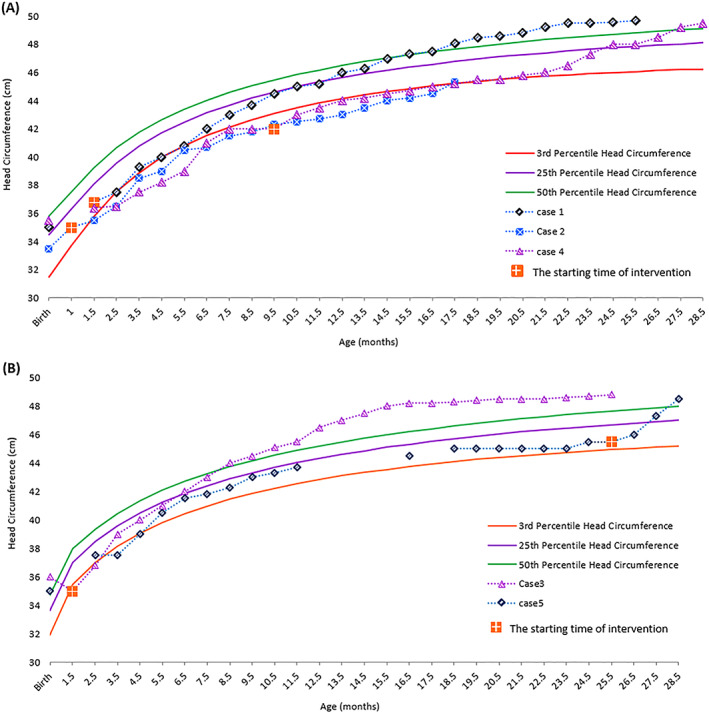
Head circumference growth trend before and after intervention according to CDC. Growth chart. (A) Boys. (B) Girls

Tables 1 and 2 present the laboratory results and details of five MSUD patients who received the Leu‐ free formula as an alternative source of Val and Iso supplementation. The plasma Leu decreased promptly within 3–4 days of starting dietary treatment. By taking 20 g/day of Xleu Maxamaid (Nutricia, https://www.nutricia.it/prodotti/xleu-maxamaid/#osmolalitagrave) in combination with BCAA‐free formula, the plasma Leu levels of Patients 1,2, 3 and 5 decreased from 1880, 1920, 1632, and 1937 μmol/L to 25, 159, 16, and 260 μmol/L, respectively, within 3 days. In Patient 4, a 9.5‐month old boy, who had prolonged high plasma Leu for more than 8 months, the plasma Leu decreased from 1520 to 19 μmol/L after receiving 15 grams/day of Leu‐free formula in addition to BCAA‐free formula for 4 days (Table 1). The head circumference in most patients (*n* = 4) improved from the 3rd percentile to more than the 50th percentile after lowering and maintaining plasma Leu over 14–32 months follow‐up (Figure 1). There was a negative correlation between head circumference and plasma Leu *p* < 0.001. All patients showed improvement in cognitive and motor development, although the rate of progress was slow particularly with walking and speech development (Table 2). Patients 2, 3, and 5 had no speech (despite speech therapy intervention) and were unable to walk unaided at the age of 17, 26, and 39 months respectively.

## 4 DISCUSSION

MSUD patients experience recurrent metabolic crises at the time of diagnosis and during fever, viral infection, illness, or constipation. If untreated, it may lead to seizures, irreversible cerebral edema, brain damage, IQ reduction, coma, and even death depending on the patient's age and severity of the disorder. The main goal of medical nutrition therapy is to reduce plasma Leu quickly to a safe therapeutic target range (75–200 μmol/L) to prevent brain damage. The present study explored an alternative source of Val and Iso to enable the lowering of plasma Leu in a short time. By temporarily using the specified amount of a Leu‐free formula (containing Iso and Val) in addition to BCAA‐free formula (given according to weight‐based requirements), this enabled a rapid reduction in plasma Leu in less than 5 days and a reversal or prevention of brain encephalopathy and cerebral edema. The present study showed that the use of BCAA‐free formula only is insufficient to reduce plasma Leu levels quickly. Before our intervention, despite receiving BCAA‐free formula, all the patients had poor metabolic control during their initial treatments, patients 3 and 4 had severe signs of Iso deficiency associated with administration of BCAA‐free formula as the only food source for more than 1 month; but their plasma Leu remained higher than 1500 μmol/L. Our results support previous studies showing that the use of BCAA‐free formula only even for a short time, causes Val and particularly Iso deficiency while plasma Leu concentrations remains elevated.^19^, ^20^, ^21^, ^22^

Following the use of this alternative Val and Iso source, most patients had significant growth in head circumference (figure) and Patients 1, 3, and 4 showed catch up growth of head circumference during follow‐up (achieving ≥50th percentile), patient 5 had a dramatic rise in head circumference (from 3rd percentile to 50th percentile) within 3‐month of follow‐up. Patient 2 who experienced respiratory arrest during hospitalization at birth, developed slowly and his head circumference remained on the third percentile despite good control of plasma Leu. The patients showed improvement in cognitive, social, emotional, and motor skills development, although there was still delay, particularly in speech development and walking (Patients 2, 3, and 5). In our study despite controlling plasma Leu, delayed myelination and periventricular white matter volume loss with thinning of corpus callosum were detected by magnetic resonance imaging (MRI) in Patients 2 and 3, respectively. Our study supports previous studies showing that the accumulation of Leu and its metabolite (aKIC) in the brain leads to neurological damage in MSUD patients at any age.^5^, ^23^, ^24^, ^25^, ^26^ Previous longitudinal studies have shown that high plasma Leu in the neonatal period and the time of diagnosis is inversely related to intellectual outcome.^4^, ^23^, ^26^ These studies showed that prevention of neurologic disturbances relies primarily on early diagnosis and prompt institution of treatment. In fact, due to the inhibitory effect of high plasma Leu and its metabolite, aKIC, on brain development, newborn screening is mandatory for early diagnosis and appropriate medical and dietary management is vital to prevent irreversible cerebral damage.^27^, ^28^ Our study showed that, using a Leu‐free formula with added Val and Iso is effective and provides a useful emergency protocol to provide prompt reduction of plasma Leu during acute metabolic crisis and prevent Val and Iso deficiency particularly for countries without accessibility to individual supplements. This approach has been used frequently during illness management and has also enabled patients to be successfully managed at home.

Based on our findings for infants aged 0–6 months, 35–59 mg/kg of Val and 35–52 mg/kg of Iso daily were sufficient during acute illness and episodes of hyperleucinemia to promote anabolism and provide immediate reduction in plasma Leu. These amounts are reduced to 15–20 mg/kg of Val and 11–16 mg/kg Iso for older infants. This is provided by using the recommended dosage of selected Leu‐free formula given in Table 3.

**TABLE 3:** Recommended dose of leucine‐free formula (used in combination with BCAA‐free formula) to reduce plasma leucine and prevent cerebral edema among MSUD patients during acute metabolic crisis

Age (month)	BCAA free‐formula^a^	Amino acids requirement		Leucine‐free formulas^b^				
		Iso (mg/kg)	Val (mg/kg)	Xleu Maxamaid (g/kg)	IVA Anamix (g/kg)	Comida Leu A formula (g/kg)	Comida Leu B formula (g/kg)	I‐valex2 formula (g/kg)
0–6	Calculates based on protein requirements	35–52	35–59	4–6	8–12	4–7	1.6–3	3.6–6
7–40		11–16	15–20	1.6–2	3–4	1.8–2.4	0.7–0.9	1.5–2

*Note*: (1) for infants more than 6 months, supplementary low protein foods such as low protein cereal, bread, pasta (made from wheat or corn starch) apple puree, and apple juice can be given in addition to the emergency protocol used in illness. (2) Leucine‐free formulae: Xleu Maxamaid and IVA Anamix (Nutricia Ltd.); Comida Leu formula A&B (Dr.Schar Deutschland GmbH); I‐valex formula (Abbot Nutrition Ltd.). (3) In this study Xleu Maxamaid was used to promote anabolism, but other alternative brands of similar formula may be used.

Abbreviations: BCAA, branched chain amino acid; Iso, isoleucine; Leu, leucine; MSUD, maple syrup urine disease; Val, valine.

^a^ BCAA‐free formula calculated according to infant's protein requirements (g/kg/day).

^b^ The leucine‐free formula is calculated based on infant's weight (g/kg) and the plasma Leu level; and added gradually (5 g/h) to prevent sudden rise of Val and Iso, the higher the plasma Leu, the more Iso and Val were needed to promote anabolism.

Our findings met the suggested daily requirement of Val and Iso intake for asymptomatic infants 0–6 month and was lower than suggested for infants older than 7 months with classic MSUD.^5^, ^8^, ^9^ It was inconsistent with previous studies that indicated that 80–120 mg/kg of Val and Iso are needed daily during acute illness.^5^, ^8^ It seems that, for nutritional management of MSUD patients during acute metabolic crisis, not only the patient weight but also plasma Leu should be considered for calculating the amount of Val and Iso supplementation required.

## 5 LIMITATIONS

We did not have mutation analysis for three patients and only studied five case studies with this form of Val and Iso supplementation, so further study is needed. The follow‐up period of these cases was variable but was over 1 year duration.

## 6 STRENGTH

This result is clinically important for the management of MSUD patients during metabolic crisis particularly when there is a no access to single Val and Iso amino acid supplements.

## 7 CONCLUSIONS

MSUD is a life‐threatening disorder, requiring rapid aggressive management to reverse catabolism. The present study suggests that temporarily giving calculated amounts of Leu‐free formula (containing Val and Iso but no Leu) as a supplement to BCAA‐free formula, will promote anabolism and aid immediate reduction of plasma Leu. This strategy helps prevent cerebral edema and irreversible brain damage particularly in case of acute metabolic crisis, or any situation that causes plasma Leu to increase rapidly.

## CONFLICT OF INTEREST

Anita MacDonald and Maryam Ziadlou have no commercial associations that might be a conflict of interest in relation to this article. We did not receive any financial support.

## ANIMAL RIGHTS

This article does not contain any studies with animal subjects.

## INFORMED CONSENT

Informed consent was obtained from parents of children included in the study. They agreed that their data could be used for scientific interest providing anonymity was maintained. We assigned unique identification number per patient to preserve data confidentially. Informed consent are available upon request.

## Data Availability

All data, laboratory results, photos, and movies of the patient's development are available with parent's permission and saved in their medical files securely.
